# Comparative Tyramide-FISH mapping of the genes controlling flavor and bulb color in Allium species revealed an altered gene order

**DOI:** 10.1038/s41598-019-48564-9

**Published:** 2019-08-19

**Authors:** Ludmila Khrustaleva, Natalia Kudryavtseva, Dmitry Romanov, Aleksey Ermolaev, Ilya Kirov

**Affiliations:** 1 Center of Molecular Biotechnology, Russian State Agrarian University-Moscow Timiryazev Agricultural Academy, 49, Timiryazevskaya Str., 127550 Moscow, Russian Federation; 2 Laboratory of Marker-Assisted and Genomic Selection of Plants, All-Russia Research Institute of Agricultural Biotechnology, 42, Timiryazevskaya str., 127550 Moscow, Russian Federation

**Keywords:** Plant genetics, Plant breeding, Chromosomes

## Abstract

Evolutionarily related species often share a common order of genes along homeologous chromosomes. Here we report the collinearity disruption of genes located on homeologous chromosome 4 in Allium species. Ultra-sensitive fluorescence *in situ* hybridization with tyramide signal amplification (tyr-FISH) allowed the visualization of the alliinase multigene family, chalcon synthase gene and EST markers on *Allium cepa* and *Allium fistulosum* chromosomes. In *A*. *cepa*, bulb alliinase, root alliinase (*ALL1*) and chalcon synthase (*CHS-B*) genes were located in the long arm but EST markers (API18 and ACM082) were located in the short arm. In *A*. *fistulosum*, all the visualized genes and markers were located in the short arm. Moreover, root alliinase genes (*ALL1* and *AOB*2*49*) showed contrast patterns in number of loci. We suppose that the altered order of the genes/markers is the result of a large pericentric inversion. To get insight into the evolution of the chromosome rearrangement, we mapped the bulb alliinase gene in phylogenetically close and distant species. In the taxonomic clade including *A*. *fistulosum*, *A*. *altaicum*, *A*. *oschaninii* and *A*. *pskemense* and in phylogenetically distant species *A*. *roylei* and *A*. *nutans*, the bulb alliinase gene was located on the short arm of chromosome 4 while, in *A*. *cepa* and *A*. *schoenoprasum*, the bulb alliinase gene was located on the long arm of chromosome 4. These results have encouraging implications for the further tracing of inverted regions in meiosis of interspecific hybrids and studding chromosome evolution. Also, this finding may have a practical benefit as closely related species are actively used for improving onion crop stock.

## Introduction

The *Allium* species are one of the few universal vegetables grown and used worldwide as an ingredient in many dishes. Moreover, the onion’s curative powers make it an important medicinal plant^1^. Onions are rich in two chemical groups that have perceived health benefits to humans: flavonoids and sulfur-containing compounds (the alk(en)yl cysteine sulphoxides, ACSOs). The enzyme alliinase operates within the biochemical pathway that produces the sulfur compounds responsible for the onion’s characteristic flavor^2^. Southern blot analysis showed that the alliinase is encoded by a gene family^3,4^. The alliinase gene from several *Allium* species, expressed in bulbs and leaves, was called ‘bulb alliinase gene’^4^. The first bulb alliinase cDNA clones of *A*. *cepa* was described by van Damme *et al*.^3^. Later, the genomic clone carrying the gene encoding bulb alliinase of *A*. *cepa* was sequenced^5^. It was found that the gene of bulb alliinase contained 4 introns and 5 exons and its size was 4015 bp (NCBI: L48614). Root alliinase gene of onion (*A*. *cepa*) was described by Lancaster *et al*.^6^. The authors identified of two isoforms (I and II) of root alliinase with molecular masses of 52.7 (Isoform I) and 50.5 (Isoform II) kD, which are differentiated by their glycosylation. The alliinase isoform II amino acid sequence has 52.8% homology with *A*. *cepa* bulb alliinase described by van Damme *et al*.^3^ Using primers designed on previously-described *A*. *cepa* alliinase EST sequence (AOB249)^7^, Lancaster and coauthors^6^ isolated a cDNA clone, which contained an open reading frame of 1493 bp (NCBI: AA451570.1) that was translated into a mature protein of 453 amino acids with high homology to root alliinase Isoform II. Later, the gene of the root alliinase isoform I (*ALL1*; NCBI: AB111058.1) was isolated and characterized in the BAC vector^4^. The 86 kb BAC clone possessed genomic DNA inserts including an 1847 bp protein-coding gene area with 5 exons and 4 introns. The amino acid sequence of *ALL1* had 65.4% identity with bulb alliinase and 67.3%. with a root alliinase Isoform II.

Our knowledge about genome evolution of the alliinase multigenes in Allium genus is scarce. Considering the fact that closely related species are actively used for improving onion germplasm^8–10^, the study of the synteny and collinearity of the alliinase genes in Allium genus is of practical interest. Effective alien introgression strategies require high rates of homoeologous recombination between host and alien chromosomes. Chromosomal structural changes impact meiotic fitness^11–13^, and the expression profile or epigenetic marks of genes near the breakpoints^14^. Thus, gene collinearity is a prerequisite for the successful introduction of new traits because meiotic recombination occurs primarily within genes^15^. A pilot genome sequencing project employing BAC and whole-genome shotgun sequencing revealed that the onion has one of the lowest gene densities found in plants^16^. In this context, the placement of genes encoding enzymes that operate on the same biochemical pathway but in different tissues is of fundamental interest for clarifying the function of biological structures and evolutionary development.

*A*. *cepa* has a huge genome of 1C = 16 Gbp^17^ which has hampered its molecular analysis. While transcriptome sequences produced from different tissues are available in the onion databases^18–20^, whole-genome sequencing is still ongoing^21^. For two decades, the main way to study the onion genome was molecular mapping. The root alliinase gene (Isoform II, AOB249) was mapped in a linkage group assigned to chromosome 4 and two loci of the bulb alliinase gene were placed in an unassigned linkage group^22^. Later McCallum *et al*.^23^ located the bulb alliinase gene in the linkage group assigned to chromosome 4. The root alliinase *ALL1* has not been mapped yet on a genetic map. A recombination map represents the linear order of genetic markers and is not directly related to the physical location of these markers along the chromosome. Thus, loci linked tightly on genetic linkage maps can be physically far apart on chromosomes and *vice versa*. Moreover, the cytogenetic map is useful in the synteny comparison between closely related species, especially for complex-genome organisms with large amounts of repetitive DNA. Despite of a high degree of genome collinearity that has been established for some plant families as the Poaceae and Solanaceae families^24^, even closely related plant species can show disruption of collinearity at the gene level^25,26^.

A fluorescence *in situ* hybridization (FISH) method was developed for visualizing a specific DNA sequence on physical chromosome^27^. However, the FISH sensitivity for detection of single copy DNA sequences was limited, especially for highly compacted plant chromosomes. An ultra-sensitive method termed tyramide-FISH (tyr-FISH) was adapted for plant cytogenetics^8^. But still there were some problems such as detection frequency of the small size of the target chromosomal DNA and precarious hybridization site for different probes^28^. Recently we developed a new approach for the probe preparation^29^ as well as a method for preparing high quality plant chromosomes. In the current study with tyr-FISH, we mapped members of the alliinase multigene family on the physical chromosomes of two widely cultivated onion crops, *A*. *cepa* and *A*. *fistulosum*, unraveling unique genome reorganization events. We report, for the first time, a various chromosomal location of genes responsible for flavor and bulb color in Allium species. We physically mapped six genes/markers in *A*. *cepa* and *A*. *fistulosum* and also established the chromosomal location of the bulb alliinase in phylogenetically close and distant species. This transgenomic cytogenetic mapping provides useful information for further study of onion genome evolution and has practical application in interspecific breeding of new onion varieties.

## Results

### Tyr-FISH direct visualization of alliinase genes on *A. cepa* and *A. fistulosum* chromosomes

The Allium alliinase gene family consists of three paralogous genes encoding two root alliinase: isoform I alliinase (*ALL1*)^4^ and isoform II alliinase (AOB249)^6^; and one bulb alliinase^3,5^. We performed PCR amplification and cloning of intron-exon parts of the genes with the length varying from 1100 to 1200 bp. The use of genomic amplicons from expressed regions that carried both exons and introns allows for increased hybridization specificity of the probes and enlarging of the target DNA sizes^29,30^. Individual clones, verified by sequencing, were labeled by Nick-translation and used for tyr-FISH experiments (see Material and Methods for details).

### Root alliinase (Isoform 1), ALL1 gene

**Figure 1:**
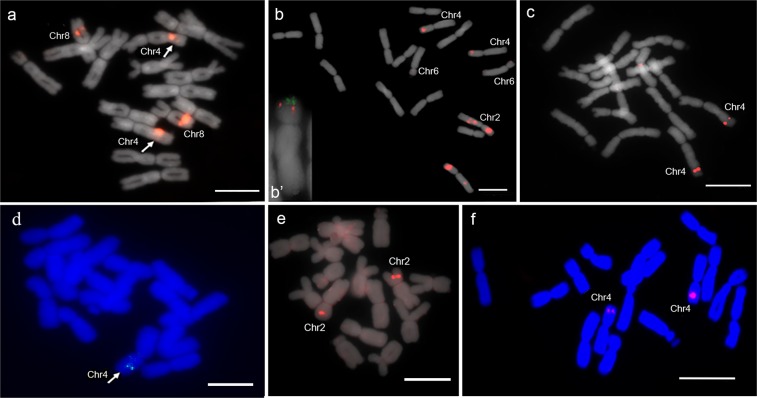
Tyr-FISH mapping of members of alliinase multigene family. Top row is metaphases of *A*. *cepa* probing with Biotin-labelled and tyramide-Cy3 visualized: (**a)** the gene encoding root alliinase Isoform I (*ALL1*), (**b)** the gene encoding root alliinase Isoform II (AOB249), (b’) extracted chromosome 6 with two-collor fluorescent signals arising from AOB249 (red signals) and 45S rDNA (green signals), (**c)** the gene encoding bulb alliinase; lower row is mitotic metaphases of *A*. *fistulosum* probing with Biotin-labelled and tyramide-Cy3 visualized: (**d)** *ALL1*, (**e)** AOB249, (**f)** bulb alliinase. *Scale bars* = 10 μm.

Tyr-FISH on *A*. *cepa* chromosomes, probing with *ALL1* gene, revealed twin-signals arising from two chromatids on the long arms of the homologous chromosomes 4 (the relative position of hybridization sites from the centromere on the chromosome arm, RPHC, - 63 ± 0.9%) and on the long arms of the homologous chromosomes 8 (RPHC - 57 ± 2.1%; Fig. 1a). The frequency of the signal detection was 50 ± 3.0% for both loci. In *A*. *fistulosum*, *ALL1* was mapped only on chromosomes 4 (Fig. 1d), and no signal was detected on chromosomes 8. In contrast to the results on *A*. *cepa*, *ALL1* was localized on the short arm of chromosomes 4 of *A*. *fistulosum* (RPHC - 60 ± 2%). The frequency of the signal detection was 48 ± 3.9%.

### Root alliinase Isoform II gene (AOB249)

To get further insight into the evolution of the alliinase gene family, we mapped the gene encoding isoform II of root alliinase (AOB249) on *A*. *cepa* and *A*. *fistulosum* chromosomes. We identified 10 AOB249 hybridization sites on three pairs of homologous chromosomes 2, 4 and 6 of *A*. *cepa* (Fig. 1b). Chromosome 2 had three AOB249 loci, namely, two loci on the long arm (RPHC - 27 ± 1.5%; detection frequency - 30 ± 2.9%; RPHC - 36 ± 1.1%; detection frequency - 42 ± 3.1%) and one locus on the short arm (RPHC - 63 ± 1.5%; detection frequency - 90 ± 1.2%). Chromosome 4 had signals in the distal position of the short arm (RPHC -72 ± 1.5%; detection frequency - 20 ± 3.2%). Chromosome 6 had signals in the distal position of the short arm (RPHC - 70 ± 1.2%; detection frequency - 28 ± 2.7%). It is known that 45S rRNA genes are located in this region of chromosome 6. To determine the position of Isoform II alliinase gene (AOB249) relatively to the rDNA loci, dual-color *in situ* hybridization was performed. It was revealed that two probes are distantly located with a more proximal position of AOB249 (Fig. 1b’). In contrast to *A*. *cepa*, the AOB249 alliinase gene was located at a single locus in the distal position of the short arm of *A*. *fistulosum* chromosome 2 (RPHC - 63 ± 1.5%; detection frequency - 80 ± 1.3%; Fig. 1e).

### Bulb alliinase gene

The bulb alliinase gene of *A*. *cepa* was localized in the distal position on the long arm of a pair of homologous chromosomes 4 (RPHC - 74 ± 1.1%; detection frequency - 90 ± 1.2%; Fig. 1c). In contrast to *A*. *cepa*, the bulb alliinase gene was localized on the short arm of homologous chromosomes 4 of *A*. *fistulosum* (RPHC - 72 ± 1.5%; detection frequency - 90 ± 1.1%; Fig. 1f).

Thus tyr-FISH mapping of three alliinase genes on *A*. *cepa* and *A*. *fistulosum* chromosomes showed contrast patterns in both chromosome location and number of loci. The results provide clear evidence for the rearrangement of chromosome 4: the root alliinase *ALL1* and the bulb alliinase genes were located on the long arm of chromosome 4 in *A*. *cepa* while in *A*. *fistulosum*, these genes were located on the short arm of chromosome 4.

### Tyr-FISH mapping of chromosome 4 to explore collinearity of genes in *A. cepa* and *A. fistulosum*

To get further insight into the extent of the rearrangement and possible disruption of collinearity, we selected additional markers: chalcone synthase (CHS-B), ACM082 and API18, which were previously genetically mapped in the linkage group assigned to chromosome 4 of *A*. *cepa*^22,23,31^.

Two markers ACM082 and API18 were distantly located from bulb alliinase on the genetic map. We applied а dual-color sequential tyr-FISH to visualize the bulb alliinase gene with each of these markers on the same metaphase.

### Chalcone synthase (CHS-B) gene

**Figure 2:**
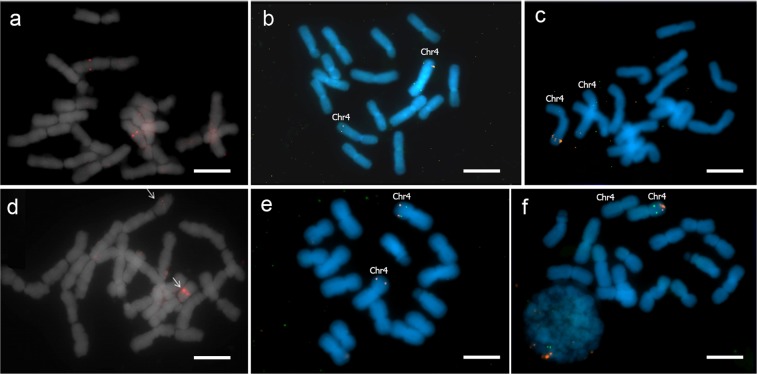
Tyr-FISH mapping of markers belonging to the linkage group assigned to chromosome 4. Top row is mitotic metaphases of *A*. *cepa*: (**a**) the gene encoding chalcone synthase (*CHS-B*), (**b**) dual-color sequential tyr-FISH probing with API18 (green) and the bulb alliinase gene (red), (**c**) dual-color sequential tyr-FISH probing with ACM082 (green) and the bulb alliinase gene (red); lower row is mitotic metaphases of *A*. *fistulosum* (**d**) the gene encoding chalcone synthase (*CHS-B*), (**e**) dual-color sequential tyr-FISH probing with API18 (green) and the bulb alliinase gene (red), (**f**) dual-color sequential tyr-FISH probing with ACM082 (green) and the bulb alliinase gene (red). *Scale bars* = 10 μm.

The physical location of CHS-B on chromosome 4 was unknown. Tyr-FISH mapping confirmed that CHS-B is located on chromosome 4 in both species, *A*. *cepa* and *A*. *fistulosum* (Fig. 2a,d). However, the position of the gene differed between the two species. The CHS-B gene was located on the long arm of chromosome 4 in *A*. *cepa* (RPHC - 53 ± 1.3%; detection frequency - 30 ± 2.8%) but on the short arm of chromosome 4 in *A*. *fistulosum* (RPHC - 50 ± 2.5%; detection frequency - 32 ± 2.5%).

### Dual-color sequential Tyr-FISH

#### ACM082

The dual-color sequential Tyr-FISH probing with the bulb alliinase gene and ACM082 revealed the ACM082 hybridization signal on the short arm of chromosome 4 in both species (Figs 2c,f, S1). In *A*. *cepa*, the position of the ACM082 signal on the short arm (RPHC) was 37.1 ± 1.4% and the detection frequency of 29 ± 3.2% while the bulb alliinase gene was distantly located on the long arm. In *A*. *fistulosum*, the RPHC was 39.6 ± 1.6% and the detection frequency was 44.0 ± 2.4%, ACM082 was located on the same arm with bulb alliinase. The ACM082 was located more proximally (closer to the centromere) compared to the bulb alliinase gene.

#### API18

The dual-color sequential tyr-FISH probing with the bulb alliinase gene and API18 revealed that the API18 cDNA clone was located on the short arm in both species (Figs 2b,e, S2). In *A*. *cepa*, the RPHC was 41.8 ± 1.3% and the detection frequency was 14 ± 2.8% while the bulb alliinase gene was distantly located on the long arm. In *A*. *fistulosum*, API18 was located on the same arm with bulb alliinase, the RPHC was 43.6 ± 2.3% and the detection frequency was 12 ± 3.2%. The chromosomal position of API18 was more distal compared to ACM082 and more proximal compared to the bulb alliinase gene. The lowest detection frequency of API18 among other probes can be explained by the fact that the API18 probe is a cDNA clone, while the other samples are genomic amplicons.

Since the detection frequency of API18 and ACM082 was relatively low, the probability of visualizing these markers simultaneously with alliinase on both homologues of chromosome 4 was small.

**Figure 3:**
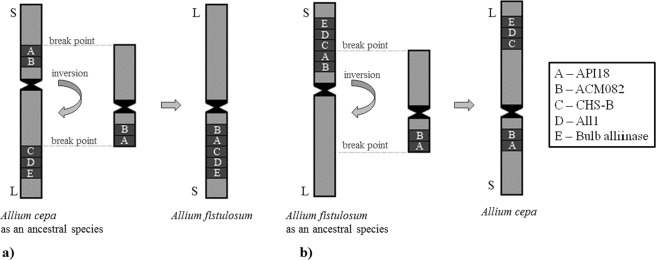
Schematic representation of the pericentric inversion of chromosome 4 in *A*. *cepa* and *A*. *fistulosum*.

Thus, based on the results of tyr-FISH mapping of the alliinase genes, chalcone synthase gene and EST markers (API18 and ACM082) in two closely related species *A*. *cepa* and *A*. *fistulosum*, we suggest that chromosome 4 containing these genes/markers underwent a pericentric inversion during the divergency of the species. This chromosomal rearrangement resulted in gene collinearity disruption between *A*. *cepa* and *A*. *fistulosum*. If we assume that the position of the visualized genes/markers in an ancestral species was similar to that of modern *A*. *cepa*, then the break point happened distal to API18 on the short arm and proximal to the CHS-B on the long arm (Fig. 3a). However, it could be that the position of mapped genes/markers in the ancestral species was similar to that of modern *A*. *fistulosum*. In this scenario the break point happened between API18 and CHS-B on the short arm and in distal/interstitial region of the long arm (3b). In both scenarios, the hypothesis might be supported by the significant differences (Wilcoxon test, p-value < 1e-5) in the centromere index between homeologous chromosomes 4 of *A*. *cepa* and *A*. *fistulosum* (*A*. *cepa* – 38 ± 0.3% and *A*. *fistulosum* – 42 ± 0.5%). A possibility of single translocation together with inversion of the locus that possessed bulb alliinase gene, *ALL1* and CHS-B or the locus with API18 and ACM082 cannot be excluded. However, in this scenario, three break points on chromosome 4 should occur. Also, it might be possible that multiple rearrangements resulting in the change of arm lengths or independent single rearrangements between species occurred.

### Detection of chromosome 4 rearrangement in other allium species

To get insight into the occurrence of the chromosome 4 inversion in other *Allium* species, we used the bulb alliinase gene as an efficient marker (detection frequency is 90%) for tyr-FISH mapping. We chose six *Allium* species with different phylogenetic distances relative to *A*. *cepa* and *A*. *fistulosum*. Of them *A*. *altaicum*, *A*. *oschaninii* and *A*. *pskemense* belong to the section Cepa (subgenus Cepa), *A*. *schoenoprasum* belongs to the same subgenus (Cepa) but another section – Schoenoprasum, A. *roylei* belongs to section Oreiprason (subgenus Polyprason) and *A*. *nutans* belongs to section Rhizirideum (subgenus Rhizirideum). Taxonomic rank is represented according to Ricroch *et al*.^17^ and Friesen *et al*.^32^.

**Figure 4:**
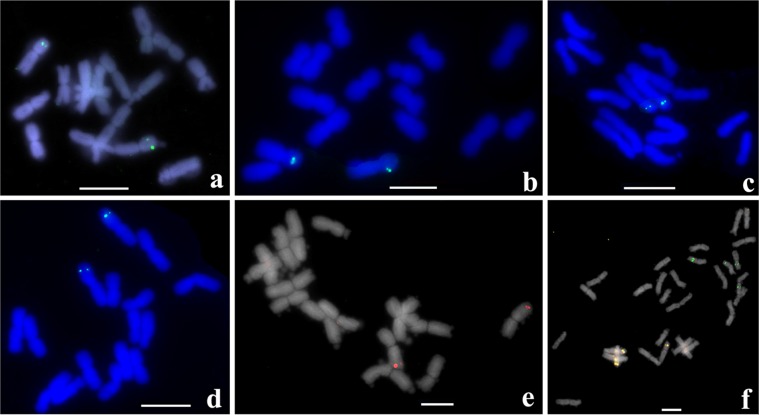
Tyr-FISH mapping of the bulb alliinase gene in Allium species: (**a)** *A*. *altaicum* (Dig-labelled, tyramide-FITC visualization, green fluorescence); (**b)** *A*. *pskemense (*Dig-labelled, tyramide-FITC); (**c)** *A*. *oschaninii* (Dig-labelled, tyramide-FITC); (**d)** *A*. *roylei* (Dig-labelled, tyramide-FITC); (**e**) *A*. *schoenoprasum* (Biotin-labelled, tyramide-Cy3 visualization, red fluorescenece); (**f)** *A*. *nutans* (bulb alliinase gene – Biotin-labelled and tyramide-Cy3; 5S  rDNA – FISH probing with Dig-labelled DNA and antiDig-FITC detection). *Scale bars = *10 μm.

**Figure 5:**
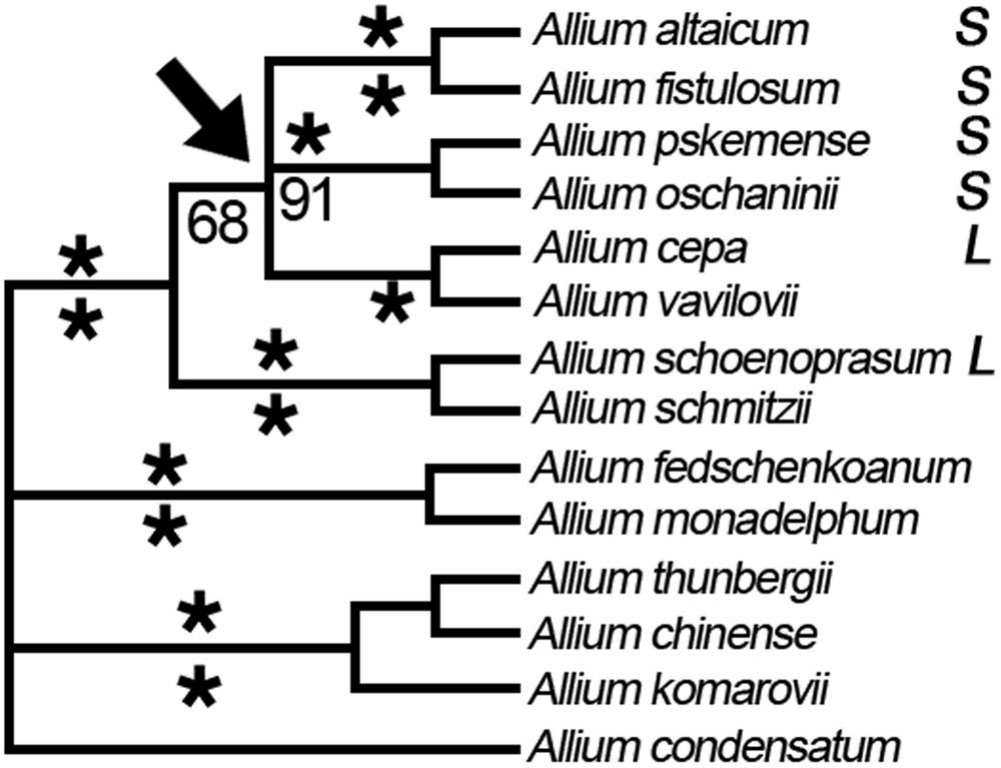
The phylogenetic tree of Allium is top part of strict consensus tree described by Friesen^32^. L - location of the gene encoding bulb alliinase in the long arm of chromosome 4; S - location of the gene encoding bulb alliinase in the short arm of chromosome 4. Arrow shows the node where the pericentromeric inversion occurred.

In *A*. *altaicum*, *A*. *oschaninii*, *A*. *pskemense*, *A*. *roylei* and *A*. *nutans*, the bulb alliinase gene was located on the short arm of chromosome 4 as in *A*. *fistulosum* while its location on chromosome 4 of *A*. *schoenoprasum* was on the long arm as in *A*. *cepa* (Fig. 4). According to the phylogenetic tree^32^ and our tyr-FISH results, the inversion of chromosome 4 occurred in ancestral species during divergence and subsequent speciation of the taxonomic clade, including *A*. *fistulosum*, *A*. *altaicum*, *A*. *oschaninii* and *A*. *pskemense*, which possessed the bulb alliinase gene on the short arm of chromosome 4 and the taxonomic clade, including *A*. *cepa* and *A*. *schoenoprasum*, which possessed the bulb alliinase gene on the long arm of chromosome 4 (Fig. 5). The position of the bulb alliinase gene on the short arm in phylogenetically distant species *A*. *roylei* and *A*. *nutans* indicates that most probably in the ancestral species, the order of the genes was as in *A*. *fistulosum*. However, to confirm this hypothesis, more *Allium* species have to be analyzed.

### Integration of chromosomal and recombination maps of the *A. cepa* chromosome 4

**Figure 6:**
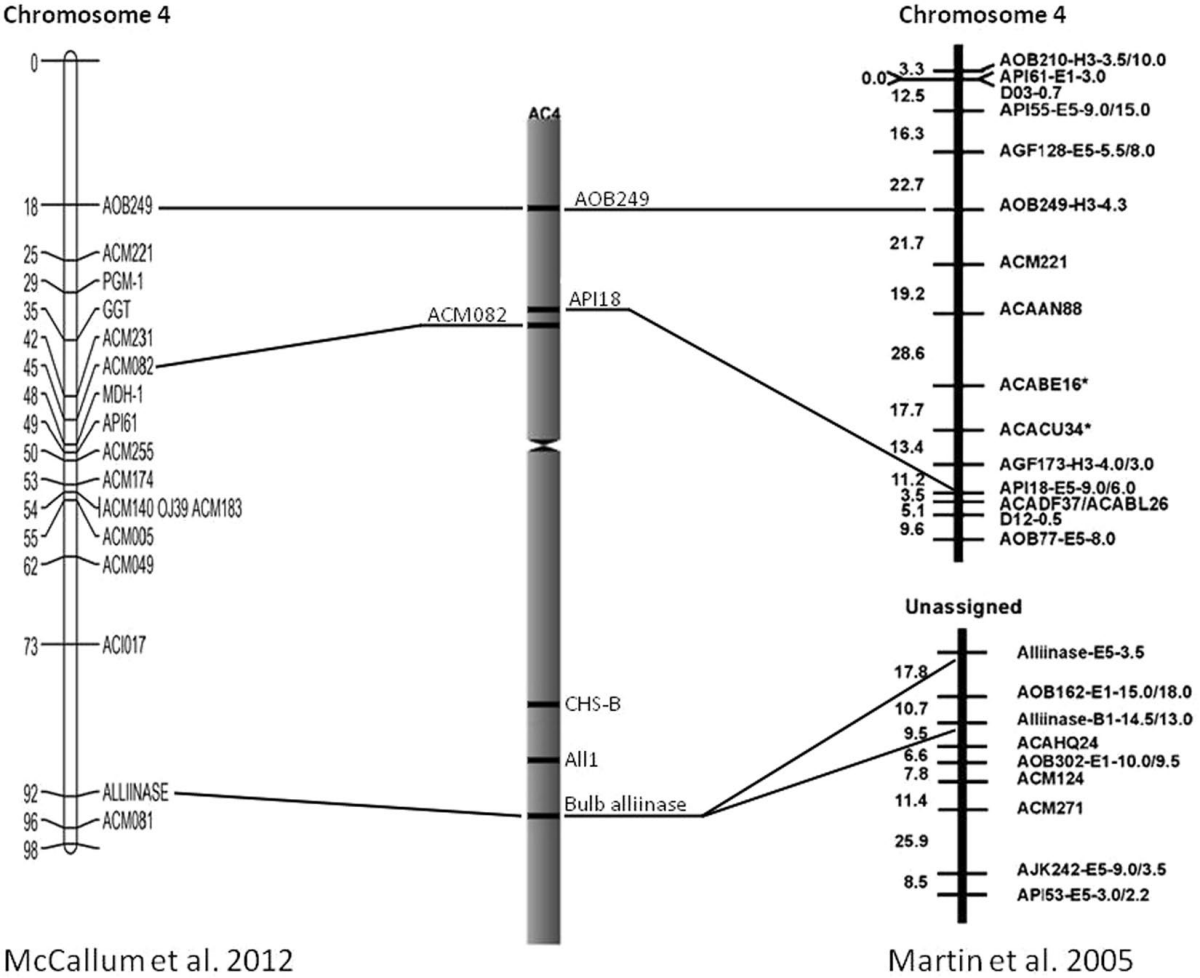
Alignment of the genetic and cytogenetic maps of *A*. *cepa* chromosome 4. The genetic map (*left figure*) is the linkage group described by McCallum *et al*.^23^ and the genetic map (*right figure*) is the linkage group described by Martin *et al*.^22^. Distances between markers are shown on the left of linkage groups in centiMorgans (cM). The relative positions (RPHC) of the tyr-FISH mapped markers/genes on the cytogenetic map (*figure in the middle*) was calculated as the ratio of the distance between the site of *in situ* hybridization and the centromere to the length of the chromosome arm.

For alignment of the position of visualized genes/markers in chromosomal and genetic maps, we used genetic maps constructed by Martin and colleagues^22^ and McCallum and colleagues^23^. The genetic map of Martin and colleagues^22^ has been developed using RFLP markers^7^ in the intraspecific onion cross ‘BYG15-23 × AC43’ and was subsequently augmented with SNP and SSR markers derived from EST sequencing. The genetic map of McCallum and colleagues^23^ has been developed using the addition of 74 co-dominant markers to the *A*. *cepa* × *A*. *roylei* interspecific map constructed primarily with dominant AFLP markers^33^. The integration of chromosomal landmarks into genetic maps of *A*. *cepa* chromosome 4 was carried out using the probes that produced reliable fluorescent signals (Fig. 6). The chromosomal map showed that the unassigned group, which possessed alliinase genes, belongs to linkage group 4 of the genetic map constructed by Martin *et al*.^22^ The presence of two loci of alliinase genes on the genetic map and only one site of probe hybridization in our chromosomal map can be explained by a high level of condensation of onion mitotic chromosomes^8^. Thus, duplicated alliinase genes could be distant from each other up to 25 Mb and still be co-localized on the mitotic metaphase chromosome. Our Tyr-FISH mapping confirmed the genetic mapping data produced by McCallum *et al*.^23^.

## Discussion

Collinearity and synteny of genes have traditionally been identified by looking for one-to-one (pairwise) conservation between species^34^. Recently, new methods for identifying genome rearrangement were developed using next-generation paired-end reads^35^ and chromosome-conformation capture sequencing (Hi-C)^36,37^. However, the above methods cannot be applied to the study of genome rearrangement in onions because the chromosome level of the whole-genome assembly is not available. Whole-genome sequencing of the onion is still ongoing^21^. Here we established the disruption of gene collinearity through visualization of genes/markers on physical onion chromosomes using the ultra-sensitive tyr-FISH method. Comparative target mapping of the alliinase genes and EST markers on the *A*. *cepa* and *A*. *fistulosum* mitotic chromosomes revealed a large ancient pericentric inversion involving the bulb alliinase gene, the root (*ALL1*) alliinase gene and the chalcone synthase gene (CHS-B). Previously performed common cytogenetic analysis, based on chromosome morphology in meiosis of hybrids between *A*. *cepa* and *A*. *fistulosum*, showed the presence of inversion loops and translocation^38–40^. These studies of meiotic chromosome structure with monochrome stain did not allow for distinguishing a particular chromosome involved in rearrangement, not to mention the mapping of genes on the physical chromosome and detection of their order disruption. Chromosomal rearrangements are thought to play an important role in speciation because they may disrupt meiosis in hybrids, thereby causing sterility^11,41–43^. An opposite-held view is that many chromosomal rearrangements are mostly incidental to speciation and have little effect on fertility^44,45^. Interspecific hybrids between *A*. *cepa* and *A*. *fistulosum* are mostly sterile^46,47^. Beyond a fundamental interest in evolutionary events causing speciation of these *Allium* species, there is applied interest in understanding the reproductive barrier between these two closely related *Allium* species. *A*. *fistulosum* is a reach source of desirable genes which are advantageous to the breeding of new bulb onion cultivars^10^. Since 1935, all subsequent attempts to transfer genes from *A*. *fistulosum* to *A*. *cepa* have failed because of sterility in backcrossed generations^46^. Could this large pericentric inversion be the cause of sterility and consequently reproductive isolation of the species? In this study, the presence of the pericentric inversion in chromosome 4 was established for *A*. *roylei* as well. The bulb alliinase gene was detected on the short arm of chromosome 4 of *A*. *roylei* as in *A*. *fistulosum*. However, *A*. *roylei* crosses readily with *A*. *cepa* and the interspecific hybrids proved to be fertile in contrast to *A*. *cepa* × *A*. *fistulosum* hybrids^9,48^. Another suggestion was put forward that nucleo-cytoplasmic incompatibility might be the cause underlying the species barrier between *A*. *cepa* and *A*. *fistulosum*^40^. Nothing is known about molecular mechanism of the reproductive barrier between these two species. While the inversion of chromosome 4 may not prevent normal meiotic behavior of chromosomes in F_1_ hybrids, relocation of the genes caused by this inversion could influence 3D genome structure resulting in changed gene expression patterns. The results obtained on Arabidopsis^49^ support this possibility. In recent years, transcriptomic analysis of gene expression based on RNA sequencing technology has become a powerful tool for unraveling the reproductive barrier mechanism between closely related plants^50,51^. Transcriptome sequences produced from different tissues are available in the onion databases^18–20^. It would be great interest to study the molecular mechanism of the reproductive barrier between *A*. *cepa* and *A*. *fistulosum* using transcriptomic analysis.

In the light of the data we obtained also the further tracing of inverted regions in meiosis of interspecific hybrids and studding chromosome evolution is of interest. It is known that recombination suppression is typically associated with inversions^52,53^. According to Rieseberg^13^, chromosomal rearrangements often suppress recombination and thereby restrict gene flow across larger genomic regions. Stevison and coauthors^12^ added to the discussion suggesting that the reduced recombination protects existing adaptive complexes and genetic incompatibilities and allows for the accumulation of additional incompatibilities between species^12^. In previous research on the second generation bridge-cross *A*. *cepa* × [*A*.*cepa* × (*A*. *roylei* × *A*. *fistulosum)*] through GISH (Genomic *in situ* Hybridization) visualization of recombination sites, a random distribution of recombination sites along the entire recombinant chromosome 4 between *A*. *cepa* and *A*. *fistulosum* was revealed^54^. However, these data do not allow any conclusions regarding the suppression of recombination in the inverted region or in the region of break points. Albini and Jones^38^ observed synaptonemal complex formation that is capable of supporting crossing over in the F_1_ hybrid between *A*. *cepa* and *A*. *fistulosum*. Also the authors reported heteromorphic bivalents and chiasma reduction in the pollen mother cells in the F_1_ hybrid, when compared with the parents. There are no data on the frequency of recombinations on a particular chromosome, as it is difficult to distinguish individual meiotic chromosomes in the onion. Future analysis of meiosis in F_1_ hybrid between *A*. *cepa* and *A*. *fistulosum* with molecular-cytogenetic markers for the pericentric inversion developed in this study will make it possible to trace the behavior of homeologous chromosome 4 providing detailed insights into how to conjugate and recombine the inverted region.

Tyr-FISH mapping of the bulb alliinase gene showed the presence of the gene on the short arm of chromosome 4 in the taxonomic clade including *A*. *fistulosum*, *A*. *altaicum*, *A*. *oschaninii* and *A*. *pskemense* and in phylogenetically distant species *A*. *roylei* and *A*. *nutans*. In two other analyzed species, *A*. *cepa* and *A*. *schoenoprasum*, the bulb alliinase gene was located on the long arm of chromosome 4. Many pericentric inversions are underdominant^13,55^, i.e., the selection against the heterozygote. Considering the heterozygote disadvantage in reproductive success, the question arises how a large pericentric inversion in Allium chromosome 4 is fixed in the population? One hypothesis is that the inversion became a hotspot for accumulating positively selected differences and genes that cause incompatibility among species^56^. An alternative hypothesis is that the inversions in fact became fixed because of local adaptation, i.e., inversions capture alleles that are adapted to the local environmental conditions^55^. Molecular, genetic and evolutionary analysis of a paracentric inversion in *Arabidopsis thaliana* showed a robust association between the inversion and fecundity under drought, and linkage disequilibrium between the inverted region and the early flowering Col-FRIGIDA allele^14^.

Mapping alliinase genes on *A*. *cepa* and *A*. *fistulosum* chromosomes revealed contrast patterns in chromosome location and a number of loci. Copy number variations play significant roles in plant evolution and adaptation. The adaptive role of the copy number amplification as a genetic mechanism by which weeds evolve resistance to herbicides was clearly demonstrated on Kochia scoparia^57^. Alliinase plays a key role in the development of onion flavor and pungency^58^. *A*. *cepa* root alliinase may have a function in sulfur assimilation and remobilization in roots^6^. Based on our *in situ* mapping results, we can only conclude the presence of duplicated DNA sequences with high similarity to the AOB249 probe in the *A*. *cepa* genome but we cannot say whether all 10 loci are functional genes. Nonetheless, considering the key role of alliinase in the onion’s characteristic flavor we may suggest that duplication of the alliinase genes contributed to this desirable trait in the long history of onion domestication.

In conclusion, we have produced rigorous evidence for the gene collinearity disruption of Allium chromosome 4 through the tyr-FISH visualization of genes/markers in two closely related species, *A*. *cepa* and *A*. *fistulosum*. Moreover, comparative transgenomic mapping of the bulb alliinase gene in phylogenetically close and distant Allium species showed a variable gene location: in *A*. *fistulosum*, *A*. *altaicum*, *A*. *oschaninii*, *A*. *pskemense*, *A*. *roylei* and *A*. *nutans* the bulb alliinase gene was located on the short arm of chromosome 4 while, in *A*. *cepa* and *A*. *schoenoprasum*, the bulb alliinase gene was located on the long arm of chromosome 4. We suppose that the altered order of the genes controlling flavor and bulb color in Allium species is the result of a large pericentric inversion. This finding points into direction that the chromosomal rearrangements may play a role in speciation and genome evolution, and may have a practical benefit as closely related species are actively used for improving onion crop stock. In spite of the great progress in bioinformatic and genomic approaches, FISH remains a ‘gold standard’ method for mapping physical chromosomes and detection their reorganization.

## Methods

### Chromosome preparation

Seeds of *A*. *cepa*. L., var. ‘Haltsedon’ (2*n* = 2*x* = 16), *A*. *fustulosum* L., var.‘Russkiy Zimniy’ (2*n* = 2*x* = 16), *A*. *schoenoprasum* L., var. ‘Medonos’ (2*n* = 2*x* = 16), *A*. *nutans* L. (2*n* = 4*x* = 32) and *A*. *altaicum* Pall., var. ‘Alves’ (2*n* = 2*x* = 16) were germinated on moist filter paper at 25 °C. Seedlings with roots about 1 cm long were pre-treated in 0.75 mM hydroxyurea for 20 h at RT and then in 0.05% colchicine for 3.5–4 h at RT. Plants of *A*. *oschaninii* O. Fedtsch(2*n* = 2*x* = 16), *A*. *pskemense* O. Fedtsch (2*n* = 2*x* = 16) and *A*. *roylei* Stearn (2*n* = 2*x* = 16) were grown in pots in a greenhouse. In order to arrest chromosomes at the metaphase stage, young roots were treated in a nitrous oxide gas chamber at elevated pressure (1.0 MPa) for 2 h and then were submerged in a saturated aqueous solution of α-bromonaphthalene (1:1000, v/v) overnight at 4 °C. The root tips were fixed in freshly prepared 3:1 (v/v) ethanol:acetic acid mixture for 1 h at RT and stored at −20 °C. Chromosome preparations were made according to the SteamDrop protocol^59^ using a cell suspension obtained after digestion of root tips in 0.1% enzyme mix (Pectolyase Y-23, Kikkoman, Tokyo, Japan; Cellulase Onozuka R-10,Yakult Co. Ltd., Tokyo, Japan and Cytohelicase, Sigma-Aldish Co.LLC, France) for 90–100 min at 37 °C.

### Isolation of genomic clones corresponding to the target genes

Genomic DNA was isolated from 5-day-old seedlings of *A*. *cepa* and *A*. *fustulosum* according to the protocol of Rogers and Bendich^60^. Primers (Table 1) were designed to produce genomic amplicons, which were cloned into the pCR2.1-TOPO vector using the manufacturer’s protocol (Invitrogene), Sanger sequenced^61^ and aligned with BLASTN to confirm identities with original NCBI sequences. The sequences of cloned genomic DNA were evaluated for repetitive elements using CENSOR^62^.

**Table 1:** Primers used for producing the genomic fragment amplifications of the target genes.

Gene	Primers 5′–3′	Accession Number in NCBI	References
Bulb alliinase	GGTCATCTCCCTTTCACCAA TTGATCAAACTCAAACGCAC	L48614.1	Gilpin *et al*.^5^
Root alliinase, Isoform I (*All1*)	GAGTGATCCGAAAGCTCCTG AGGTTCACACATGCGCAATA	AB111058.1	Do *et al*.^4^
Root allinase, Isoform II (AOB249)	CAGTTGGCAATGCTAAAGTC TCCCACTCACATTTCAACCA	AF126049.1	Lancaster *et al*.^6^
Chalcon synthase (CHS)	TCCATGATCAGGAAACGCTAC CGAACGCACCCATTAACAAT	AY221245.1	Kim *et al*. submitted to NCBI in 2003
АСМ082	GTGCAGTTGGAGATGGTGTG TGTGAATGTACCGCTCGTACC	CF436620.1	McCallum *et al*.^23^

The bulb alliinase gene primer set was designed on a region including two introns (2 and 3) and three exons (2, 3 and 4) of the genomic DNA clone (L48614.1) PCR with the bulb alliinase primers and genomic DNA of *A*. *cepa* and *A*. *fustulosum*, resulting in a 1100 bp PCR product for both species. BLASTN sequence analysis of clones possessing the 1100 bp bulb alliinase gene fragments from *A*. *cepa* showed identity with the nucleotide sequence of the bulb alliinase gene of *A*. *cepa* (L48614. 1). BLASTN analysis of the clones possessing PCR products of *A*. *fistulosum* revealed a 91% identity with the sequence of bulb alliinase of *A*. *cepa* (L48614.1).

The root alliinase Isoform I (*ALL1*) primer set was designed on a region including exons2-exon5 – 3′UTR of the genomic DNA clone (AB111058.1). PCR, with the *ALL1* alliinase primers and genomic DNA of *A*. *cepa* and *A*. *fustulosum*, resulted in a 1200 bp PCR product for *A*. *cepa* and *A*. *fistulosum* species. BLASTN sequence analysis of clones possessing PCR products of *A*. *cepa* and *A*. *fistulosum* revealed the highest similarity to the *ALL1* sequence (AB111058.1).

The root alliinase Isoform II (AOB249) primer set was designed using mRNA sequence identical to AOB249 (AF126049.1). PCR, with the AOB249 alliinase primers and genomic DNA of *A*. *cepa* and *A*. *fustulosum*, resulted in a 1200 bp PCR product for both species. BLASTN sequence analysis of clones possessing the PCR products revealed identity with the original sequence (AF126049.1).

The chalcon synthase (CHS-B) primer set was designed on a region of cDNA sequence of *A*. *cepa* (AY221245.1). PCR, with the CHS-B primers and genomic DNA of *A*. *cepa* and *A*. *fustulosum*, resulted in a 1000 bp PCR product for both species. BLASTN sequence analysis of clones possessing PCR products confirms their identity with the sequence of the cDNA of *A*. *cepa* (AY221245.1).

The ACM082 primer set was designed on a region mRNA sequence of *Allium cepa* cDNA clone ACACY23 (CF436620.1). PCR, with the designed primers and genomic DNA of *A*. *fistulosum*, resulted in a 1100 bp PCR product. BLASTN sequence analysis of clone possessing PCR product confirm his identity to the sequence of cDNA of *A*. *cepa* (CF436620.1).

The API18 (AA451545) cDNA clone was kindly provided by Professor Michael Havey from the University of Wisconsin-Madison University. The plasmid DNA of API18 cDNA clone was labeled by nick-translation and used as a probe in the Tyr-FISH experiment.

A pTa71 plasmid that includes a 9 kb EcoRI fragment of the rDNA unit from wheat (*Triticum aestivum*) was used as a probe for visualization of 45S rRNA genes^63^.

### Tyr-FISH and FISH

DNA probes were labeled with digoxigenin-11-dUTP or biotin-16-dUTP using DIG- or Biotin-Nick Translation Mix (Roche, Mannheim, Germany). The chromosome slides were dried overnight at 37 °C and pretreated with 4% paraformaldehyde in 2 × SSC followed by dehydration in 70%, 90%, and 100% ethanol. The hybridization mixture consisted of 50% (v/v) deionized formamide, 10% (w/v) dextran sulfate, 2 × SSC, 0.25% sodium dodecyl sulfate, 1.25 ng/μl probe DNA. The hybridization mix was denatured at 75 °C for 5 min, subsequently placed on ice for 5 min, and added to the chromosome slides. Slides were then denatured for 5 min at 80 °C and hybridization was carried out at 37 °C overnight. Post-hybridization washing was performed in 50% (v/v) formamide in 2x SSC for 15 min at 42 °C. Tyramide-detection of unique DNA sequences (alliinase genes, *CHS-B*, API18, ACM082) was performed as described by Khrustaleva *et al*.^30^ 45S r RNA genes were detected using common FISH with anti-DIG-FITC according to Kirov *et al*.^64^ Chromosomes were counterstained in 1.5 μg/ml DAPI in Vectashield anti-fade (Vector Laboratories, http:// www.vectorlabs.com).

### Dual-color sequential Tyr-FISH

DNA probe of bulb alliinase was labeled with biotin-16-dUTP and was detected with streptavidin-HRP and Tyramide-Cy3 (red fluorescence) and DNA probes of API18 and ACM082 were labeled with digoxigenin-11-dUTP and were detected with Anti-DIG-POD and Tyramide-FITC (green fluorescence). The hybridization mixture consisted of 50% (v/v) deionized formamide, 10% (w/v) dextran sulfate, 2 × SSC, 0.25% sodium dodecyl sulfate, 1.25 ng/μl probe DNA of bulb alliinase and 1.25 ng/μl probe DNA of API18 or ACM082. Pretreatment, hybridization and post hybridization stringency washing were as described above. The remaining HRP activity after the first detection with Tyramide-Cy3 was deactivated by adding 100 μl of 3% H_2_O_2_ in TNT (0.1 M Tris-HCl, pH 7.5, 0.15 M NaCl, 0.05% Tween 20) for 30 minutes.

Slides were examined under a Zeiss AxioImager M1 microscope (http://www.zeiss.com). Selected images were captured using a digital AxioCam camera (http://www.zeiss.com). Image processing and thresholding were performed by AxioVision v.4.6 image analysis software. The captured images of the chromosomes and position of tyr-FISH signals were measured using the program DRAWID^65^. Only non-overlapping chromosomes were used for measurements of positions of tyr-FISH signals. The relative position of hybridization sites on chromosomes (RPHC) was calculated as the ratio of the distance between the site of hybridization and the centromere to the length of the chromosome arm. Karyotype analysis and identification of individual chromosomes with fluorescent signals were performed according to bulb onion nomenclature^66^ and previously published karyotypes of closely related *Allium* species^67^.

## Supplementary information

Fugures S1, S2
