# Supplementary material for: Comparative Tyramide-FISH mapping of the genes controlling flavor and bulb color in Allium species revealed an altered gene order
Source: Sci Rep. 2019 Aug 19;9:12007. doi: 10.1038/s41598-019-48564-9 (PMC6700127; doi:10.1038/s41598-019-48564-9)
Supplement: Supplementary file 1 — Fugures S1, S2 [file 41598_2019_48564_MOESM1_ESM.pdf]

Supplementary information

Comparative Tyramide-FISH mapping of the genes controlling flavor and bulb color in Allium species revealed an altered gene order

Ludmila Khrustaleva[1,*], Natalia Kudryavtseva[1], Dmitry Romanov[1], Aleksey Ermolaev[1], Ilya Kirov[1,2]

[1]Center of Molecular Biotechnology, Russian State Agrarian University-Moscow Timiryazev Agricultural Academy, 49, Timiryazevskaya Str., 127550 Moscow, Russian Federation

[2]Laboratory of Marker-Assisted and Genomic Selection of Plants, All-Russia Research Institute of Agricultural Biotechnology, 42,Timiryazevskaya str., 127550 Moscow, Russian Federation

Corresponding author:*ludmila.khrustaleva19@gmail.com

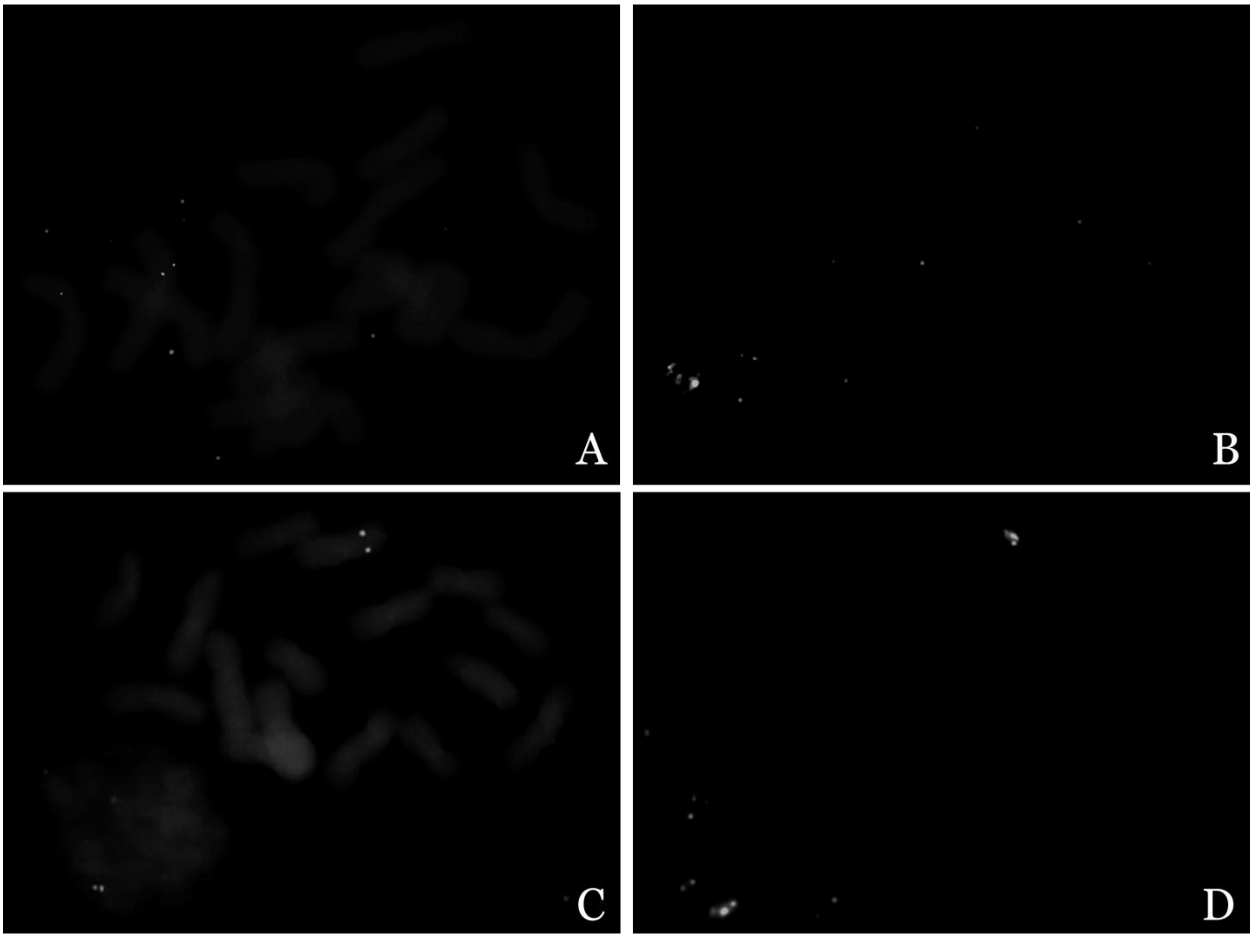

Figure S1 Dual-color sequential tyr-FISH mapping of ACM082 on chromosome 4 of ***A. cepa***: **A** – ACM082, FITC filter image; **B –** bulb alliinase gene, Cy3 filter image; ***A. fistulosum*: C**–ACM082, FITC filter image; **D -** bulb alliinase gene, Cy3 filter image

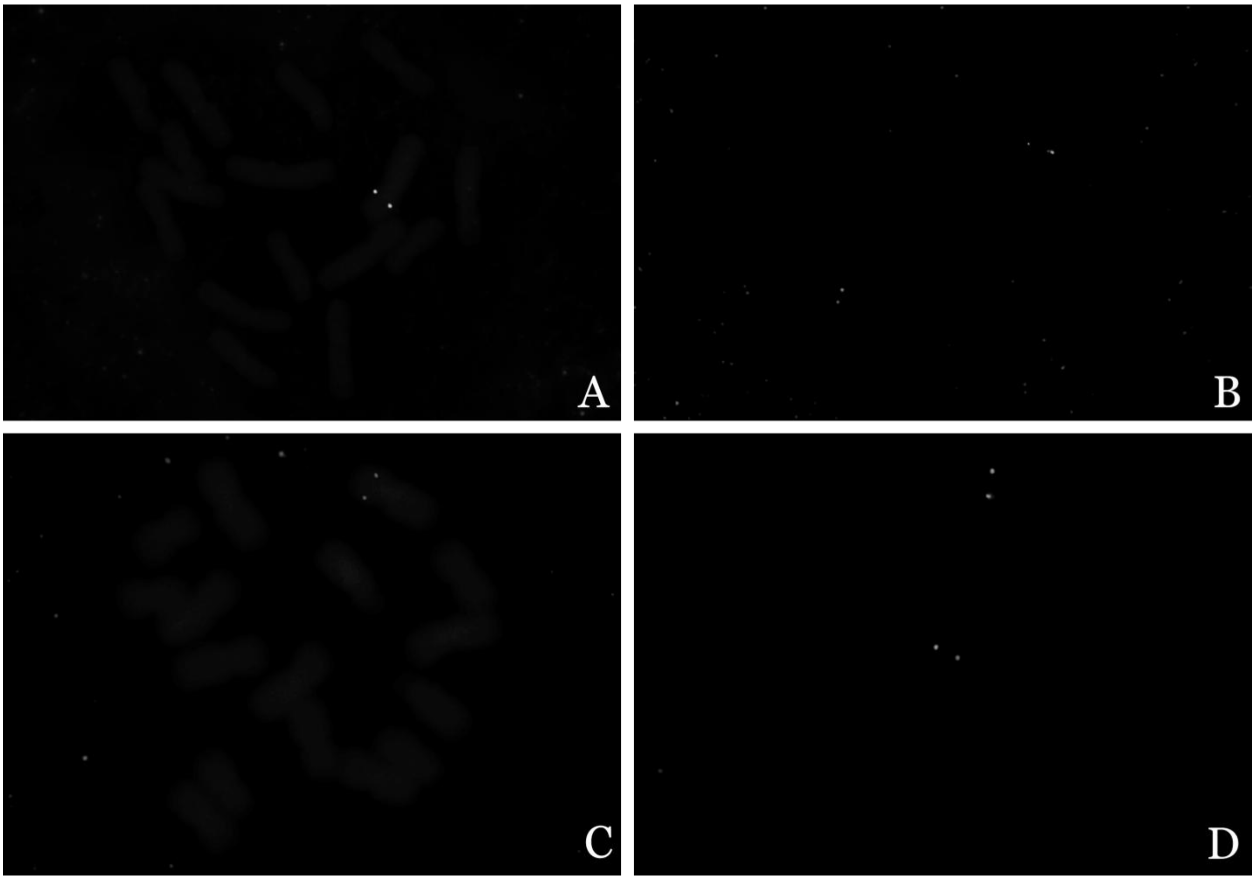

Figure S2 Dual-color sequential tyr-FISH mapping of API18 and bulb alliinase gene on chromosome 4 of ***A. cepa***: **A** – API18, FITC filter image; **B –** bulb alliinase gene, Cy3 filter image; ***A. fistulosum***: **C**–API18, FITC filter image; **D -** bulb alliinase gene, Cy3 filter image
